# *In vitro*, *ex vivo* and *in vivo* antihypertensive evaluation of enzymatic hydrolysates of Californian red worm (*Eisenia fetida*) proteins

**DOI:** 10.1016/j.heliyon.2024.e25715

**Published:** 2024-02-02

**Authors:** Yhoan S. Gaviria G, Carlos M. Guerra, José E. Zapata M

**Affiliations:** a Nutrition and Food Technology Research Group, Universidad de Antioquia, calle 70 No. 52-21, Medellín, Colombia; b Grupo de investigación GIRYSOUT, Universidad del Tolima, Ibagué, Colombia

**Keywords:** Antihypertensive, Peptide, Aorta, Antioxidant, Amino acid, Systolic pressure

## Abstract

Hypertension is an important risk factor concomitant with cardiovascular disease (CVD) states, which is why we set out to evaluate Californian red worm hydrolysates on antihypertensive activity both *in vitro*, *ex vivo*, using rabbit aortic rings and *in vivo* using hypertensive induced rats. The worms were manually separated, washed with water, purged for 4 h with 4 % sodium bicarbonate, sacrificed with 7 % saline, and finally washed with drinking water. The *in vitro* antihypertensive capacity was performed by measuring angiotensin-converting enzyme inhibition; for the *ex vivo* assays, rabbit aorta was used to measure relaxation; for the *in vivo* assays, rats with induced hypertension were used to perform acute (hypotension) and chronic assays, using captopril as a control in all assays. With respect to angiotensin-converting enzyme (ACE) inhibition, the EC50 value of the worm hydrolysate was found to be 358 ppm; with respect to the analysis in aortic rings, it was found that the mechanisms of action of the hydrolysate are endothelium-dependent, presenting a maximum relaxation of 35 %. With respect to the *in vivo* assays, the hypotensive test showed that the hydrolysate can reduce blood pressure by up to 32 % in only 2 h, while the chronic analysis showed that the hydrolysate at 150 ppm did not present statistically significant differences with the control (captopril) during the 15 days of analysis. The Red Californian earthworm hydrolysate presents bioactive compounds identified with antihypertensive activities *in vitro*, *ex vivo* and *in vivo* in different isolated and animal models. The study demonstrates the efficacy of the hydrolysate to be used as an alternative in the treatment and prevention of hypertension, and it can be implemented in functional foods or nutraceutical foods. Antihypertensive peptides, particularly those that inhibit angiotensin-converting enzyme (ACE), hold significant importance in medical research, specifically in the context of cardiovascular disease treatment, particularly hypertension. The focus on these peptides and the potential implications of their results in medical research can be summarized through several key points: 1) Mechanisms of Action-Antihypertensive peptides function by inhibiting ACE or renin, crucial enzymes in blood pressure regulation. 2)Alternatives to Synthetic Drugs, 3) Additional Health Benefits, and various other factors.

## Graphical abstract

**Figure:**
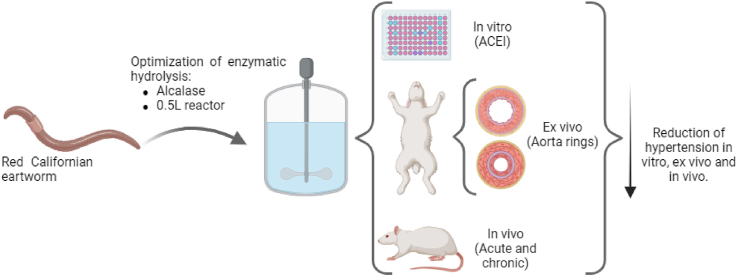

## 1 Introduction

The Red Californian earthworms (*Eisenia Foetida*) are a high-quality protein source that can be used in human and animal nutrition [1]. Although they are primarily known for their use in the production of organic fertilizers [2], they are also considered a valuable source of biologically and pharmacologically active compounds for the treatment of various diseases [3]. Since ancient times, they have been used for therapeutic purposes, especially in Chinese and Hindu cultures [4]. The use of various species of earthworms has been recorded throughout history. Approximately five hundred years ago, Shizhen Li compiled the famous book “Compendium of Materia Medica,” which described earthworms as prescription drugs to treat fever and as diuretics in the form of dried powder, a practice that is still in use today [5]. In recent decades, with the advance of biochemical technology, research has begun to be conducted on the pharmacological effects of compounds extracted from various earthworm species [6]. These organisms are nutritionally dense [4] and possess antipyretic, antispasmodic, detoxifying, diuretic, hypotensive, antiallergic, antiasthmatic, antioxidant, antimicrobial, anticancer, anti-inflammatory, and antiulcer properties [7].

Proteins perform the most functions in all living cells, from the basic structural organization of the cell to metabolic and regulatory functions [8]. As a result, the global demand for high-quality proteins is ever-increasing, both for animal feed and human consumption [9]. Moreover, proteins are a substrate for enzymatic hydrolysis, from which bioactive peptides and other products can be obtained [10]. This process involves the cleavage of a peptide bond through the action of water and enzymatic or chemical catalysis [11]. The molecular properties of proteins change due to hydrolysis, producing fragments of a lower molecular weight, increased charge, and the release of hydrophobic groups, among others [12]. These changes improve the nutritional value and functional properties of proteins (texture or flavor), either by reducing allergenic compounds or generating bioactive peptides. Enzymatic hydrolysis is the most widely used technique for producing bioactive hydrolysates [13], as it involves moderate operating conditions with some substrate specificity [14]. Bioactive peptides can elicit positive physiological responses beyond their basic nutritional functions in the supply of nitrogen and essential amino acids. These peptides are encrypted in the original protein sequence and are only expressed and released from the protein upon cleavage of the peptide bonds. The modern lifestyle, rife with stress and anxiety, has led to an increase in conditions like hypertension, commonly known as High Blood Pressure (HBP). This condition can be classified as low, moderate, or high. Hypertension is diagnosed when blood pressure consistently exceeds 140 over 90 mmHg. This means a systolic pressure (the first number) above 140 and a diastolic pressure (the second number) above 90. The thresholds for diagnosis are set at 140 mmHg or higher for systolic blood pressure (SBP) and 90 mmHg or higher for diastolic blood pressure (DBP) [15]. When any component of the integrated neurohumoral system, which regulates blood pressure, is compromised or malfunctions, it disrupts the equilibrium, potentially leading to hypertension and associated comorbidities. To prevent or delay the onset of hypertension, research has led to the development of various antihypertensive drugs. These include ACE inhibitors, which block the enzyme responsible for producing angiotensin II, a substance that narrows blood vessels. Angiotensin II receptor blockers (ARBs) also target this pathway but by blocking the receptors for angiotensin II. Dihydropyridine calcium-channel blockers work by relaxing the blood vessels, and thiazide diuretics help in reducing blood pressure by removing excess fluid and salt from the body [16]. *In vitro* angiotensin-converting enzyme (ACE) inhibitory peptides are among the most researched peptides [[17], [18], [19]]. ACE cleaves angiotensin I into angiotensin II, a potent vasoconstrictor that is a significant factor in hypertension [20]. Due to this, ACE inhibitors have been widely developed to prevent the production of angiotensin II and the triggering of cardiovascular disease. They are the starting point for developing *in vivo* antihypertensive substances [21]. This is greatly significant, as hypertension is a major concomitant risk factor for states associated with cardiovascular disease (CVD), such as coronary heart disease, peripheral artery disease, stroke, and kidney disease. Essential hypertension, the most common type of hypertension (90–95 % of cases), appears as an increase in an individual's blood pressure due to an unknown cause [22].

On the other hand, there is a close link between the inhibition of hypertension and the presence of antioxidants [20,23]. This is because oxidative stress causes a decrease in available nitric oxide (NO), which leads to endothelial dysfunction and a decrease in vasodilation, in addition to the inhibition of the nitric oxide synthase enzyme (NOSe). Superoxide is produced instead of NO, thus maintaining oxidative stress [24]. ACE inhibitors are particularly attractive in this context since they increase nitric oxide (NO) bioactivity and decrease the generation of Ang II (vasoconstrictor agent). This has a favorable effect on the redox potential of the vascular system, contributing to the stability of the atherosclerotic plaque. In this regard, antioxidant activity has been reported in enzymatic hydrolysates derived from *Eisenia fetida* proteins, so it is to be expected that these hydrolysates also present antihypertensive activity [25,26]. This study evaluated the *in vitro*, *ex vivo*, and *in vivo* antihypertensive activity of *E. fetida* protein hydrolysates.

## 2 Materials and methods

### 2.1 Reagents and chemicals

The reagents *o*-phthalaldehyde (OPA), chlorohydric acid (37 %), phenol (>99 %), 29-fluorenylmethyl chloroformate (FMOC), mobile phase (methanol, acetonitrile), hippuryl-L-histidyl-L-leucine (HHL), and ACE (ACE 3.4.15.1, 5.1 U/mg) were obtained from Sigma-Aldrich (Oakville, Ontario, Canada) and carbamoylcholine chloride (212385-M), phenylephrine hydrochloride (Pg126), N-nitro-L-arginine methyl ester hydrochloride (N5751, L-NAME) were obtained from Sigma-Aldrich (St. Louis, MO, USA). Alcalase® 2.4 L (commercial protease obtained from fermentation of Bacillus licheniformis, non-specific serine endopeptidase) was supplied by Novozymes (Bagsværd, Denmark). All reagents implemented in this study were analytical grade.

### 2.2 Handling of raw materials

We manually separated the Red Californian earthworms (*Eisenia fetida*) from their feeding substrate, washed them with potable water to remove the substrate residues, purged them for 4 h with a 4 % sodium bicarbonate solution, and then washed them with potable water to remove the bicarbonate residues. The earthworms were then sacrificed by placing them in a 7 % saline solution for 30 min. Finally, we washed the paste with potable water, and it was frozen for later assays.

### 2.3 Amino acid analysis

*E. fetida* was subjected to acid hydrolysis using a 6 % HCl and 0.1 % phenol solution. This hydrolysis was conducted at 110 °C for 16 h. Prior to precolumn derivatization, the samples were derivatized using *o*-phthalaldehyde (OPA) for the primary amino acids and 9-fluorenylmethyl chloroformate (FMOC) for the secondary amino acids [27]. The amino acids were then analyzed using an UltiMate 3000 HPLC system (Thermo Fisher Scientific, United States) equipped with a UV/Vis detector and a diode array detector (DAD). The analysis used a 5-μm analytical column, specifically the 4.6 × 75 mm ZORBAX Eclipse AAA-C18 (manufactured by Agilent, United States). The mobile phase had the following composition: water, methanol, acetonitrile (solvent B), and a NaH_2_PO_4_ 40 mM buffer solution with a pH of 7.8 (solvent A), a gradient of 0–100 % in 25 min of solvent B with a flow rate of 1.5 mL/min was used [27].

### 2.4 Enzymatic hydrolysis

The enzymatic hydrolysis was performed in a 0.5L reactor full of the working solution. Temperature (45 °C) and pH (8.5) were monitored with a glass combination electrode connected to a TitroLine 6000 automatic titrator (SI Analytics GmbH, Germany). The reaction was monitored for 4000 s based on the degree of hydrolysis (DH), defined as the ratio of the number of hydrolyzed peptide bonds at a given time (h) to the total number of peptide bonds in the native protein per unit weight (ht). Ht is calculated by adding the total moles of amino acids within the substrate [28]. The DH was determined using proton titration or pH-static titration, which works as follows: as hydrolysis progresses in an alkaline medium, the terminal carboxyl group dissociates completely, and the released protons are distributed according to the protonation equilibrium of the released α-amino groups, thus presenting a decrease in pH [29]. The required number of moles of base to keep a constant value is equal to the moles of the cleaved peptide bonds; in this case, we used a NaOH 1 N solution. We implemented Equation (1) to calculate the DH [28].

(1) $$DH\;\left(\%\right)=\frac{B*N_{B}}{M_{P}*\propto *h_{t}}*100$$

B corresponds to the volume of consumed sodium hydroxide in L. MP is the mass of protein loaded into the reactor in kg. N_B_ represents the normality of sodium hydroxide, and α is the degree of dissociation of the amino groups released on hydrolysis. The value of α is calculated based on the temperature and pK value of the reaction, using Equations (2), (3)), respectively [29]. Where pK corresponds to the equilibrium constant of the reaction, which depends on the temperature (T) in kelvin (equation (3)).

(2) $$\propto =\frac{10^{pH-Pk}}{\left(1+10^{pH-Pk}\right)}$$

(3) $$pK=7,8+\frac{\left(298-T\right)}{298*T}*2400$$

### 2.5 Evaluation of angiotensin-converting enzyme (ACE) inhibitory activity

We implemented the method used by Cushman & Cheung (1971) with some modifications [30,31]. We added 40 mL of each sample to 100 mL of HHL substrate (hippuryl-L-histidyl-L-leucine) in a 0.1 M sodium borate buffer solution with 0.3 sodium chloride at a pH of 8.3. Subsequently, we added 2 mU of the ACE (ACE 3.4.15.1, 5.1 U/mg) (Sigma; Germany), which was dissolved in 50 % glycerol. The reaction was run at 37 °C for 30 min. We lowered the pH by adding 150 mL of 1 N HCl to inactivate the enzyme and the resulting hippuric acid was extracted with 1000 mL of ethyl acetate. 750 mL of the organic phase was collected after stirring and subsequent centrifugation at 4000×*g* for 10 min at room temperature. This volume was evaporated by heating it at 95 °C for 15 min. The hippuric acid residue was dissolved in 800 mL of distilled water. After stirring, absorbance was measured at 228 nm.

ACE inhibitory activity is calculated using Equation (4), while the EC_50_ value is calculated as the amount of soluble protein required to inhibit 50 % of the enzyme. The activity of each sample was determined in triplicate. We used Equation (4) to calculate the inhibitory activity of each sample.

(4) $$Inhibitory\;activity\;\left(\%\right)=\frac{{Abs}_{Control}-\;{Abs}_{sample}}{{Abs}_{Control}-\;{Abs}_{blank}}*100$$

${Abs}_{Control}$ represents the absorbance of hippuric acid after ACE action without inhibitors. ${Abs}_{blank}$ corresponds to the absorbance of unreacted hippuryl-L-histidyl-L-leucine (HHL) extracted with ethyl acetate. ${\;Abs}_{sample}$ is the absorbance of the hippuric acid formed after ACE action in the presence of inhibitory substances.

### 2.6 Ethics statement

The Ethics Committee for Animal Experimentation of the University of Antioquia in the minutes of session number 136 (extraordinary) of November 17, 2020, and the Bioethics Committee of the University of Tolima in session number 06 of 2019 granted approval for the experiments performed in this manuscript.

### 2.7 *Ex vivo* analysis

The rabbits were anesthetized with intraperitoneal sodium thiopental (60 mg/kg) and then euthanized by cervical dislocation. The descending aorta was rapidly dissected without branches and was preserved at 4 °C in Krebs–Henseleit solution (KH) with the following composition (mM): NaCl 118; KCl 4.7; KH_2_PO_4_ 1.2; MgSO_4_ 1.2; NaHCO_3_ 25.0; glucose 11.1; CaCl_2_ 2.5; pH 7.4. Excess perivascular tissue was removed from the aortic segment under a stereoscope and was then cut into 3–5 mm rings. The rings were used for relaxation and contraction experiments within the first hour after euthanasia [32,33]. The rings were placed in a PanLab LE 13206-organ bath (Barcelona, Spain) with 10 mL of KH solution at 37 °C and were bubbled with carbogen. The contractile response was recorded using a PowerLab PL-3504 (ADInstruments; Spain) and was analyzed with the LabChart 8 Dose Response Software Module (ADInstruments; Spain). All rings were stabilized at a resting tension of 2 g for 90 min before agonist stimulation. During this period, the KH solution was replaced every 20 min. After stabilization, each arterial ring was stimulated twice with 40 mM KCl for 20 min. They were washed between each stimulation. Then, for all arterial rings, 1 μM phenylephrine (PE) and 10 μM CCh were consecutively added to the bath to confirm the presence of endothelium. For studies requiring an intact endothelium, the ring was discarded if the relaxation resulting from the carbamylcholine (CCh) was not 70 % or more. In experiments requiring the absence of functional endothelium, the aortic rings were denuded by gently rubbing the lumen with closed micro forceps. The procedure was considered successful if the response to CCh was suppressed. We then conducted the following evaluations.

Protocol #1. Blocking effect of *E. fetida* peptides (LP) on contractile agonists. Intact rings were preincubated with LP at a single concentration of 100 μM for 20 min. The rings were then separately exposed to angiotensin II (Ang II) at concentrations from 0.1 nM to 100 nM.

Protocol #2. Relaxation studies were performed after inducing contraction with 1 μM PE. Concentration-response curves were generated by exposing aortic rings with endothelium (intact), without endothelium (denuded), or with endothelial blockage (using L-NAME 100 μM) to cumulative concentrations of LP (0.1–1000 μM) [32,33].

### 2.8 *In vivo* acute hypotensive analysis

For the acute *in vivo* assay, we used 8-week-old Wistar rats weighing between 250 and 300 g (n = 5 per treatment). We used orally administered water for the blank control and intraperitoneal captopril at 5 mg/kg (Sigma-Aldrich) for the positive pharmacological reagent control.

The hypotensive effect was evaluated with the *E. fetida* hydrolysate, which was administered orally with a feeding tube at concentrations of 75 mg/kg and 150 mg/kg. Dose selection was based on Fu et al., 2020 [32] and preliminary experiments. The animals' blood pressure and heart rate were measured indirectly with a CODA tail blood pressure monitor (Kent Scientific, USA). The animals were previously warmed up so their blood flow could be sufficient for the pressure reading. Initially, the animals were adapted to the experimental environmental conditions of the trial by receiving a standard ad libitum diet. After the adaptation period, the device was placed on the animals’ tails, which, once insufflated, allowed us to take the blood pressure measurements. The basal systolic pressure and heart rate values were measured before and after administering the hydrolysate, taking measurements every 30 min for 150 min.

### 2.9 *In vivo* chronic antihypertensive analysis

We used 8-week-old Wistar rats weighing between 250 and 300 g (n = 5 per treatment). The animals were kept in a 7-day adaptation period under the environmental conditions of the study and received a standard ad libitum diet. Subsequently, L-NAME (50 mg/kg/day) was administered for one week to induce hypertension. Next, captopril (5 mg/kg) was administered as a positive control, and the *E. fetida* hydrolysate was administered as the trial antihypertensive drug at two concentrations (75 and 150 mg/kg) every 48 h. Dose selection was based on Fu et al., 2020 [32] and preliminary experiments. We measured the blood pressure by placing the device on the animals’ tails and after insufflating the device. The basal systolic pressure and heart rate values were measured the day after administering the controls and the hydrolysate to avoid false positives.

### 2.10 Statistical analysis

All data are presented as means ± SEM from three to five animals for each treatment group. The data collected was evaluated at a confidence level of 95 % by means of the hypothesis test to determine the difference in means using Fisher's LSD (Least significant difference) test with statgraphics centurion XVI software and a *p*-value <0.05 was considered statistically significant.

## 3 Results and discussion

### 3.1 Amino acid analysis and *in vitro* antihypertension analysis

Table 1 shows the amino acid composition of *E. fetida*. The table shows a high concentration of glycine, histidine, leucine, lysine, and glutamic acid. These results are similar to those reported by Garczyńska et al. (2023) a study evaluating the nutritional composition of earthworms as a protein source [34]. Additionally, the presence of a high content of essential amino acids (EAA: 54 %) suggests the high nutritional value of this protein. Further, a high number of hydrophobic amino acids (HAA: 25 %) can be observed, which have been reported as being excellent angiotensin-converting enzyme inhibitors (ACEI), mainly because the hydrophobic amino acids located in the active site of the enzyme allow the interaction of uncharged amino acids [[35], [36], [37]].

**Table 1:** Relative concentration of amino acids.

Amino-acid	Abbreviation	Concentration (mg/100 g)
Aspartic acid	ASP	291.91
Glutamic acid	GLU	694.14
Asparagine	ASN	31.78
Serine	SER	470.90
Histidine	HIS	794.15
Glycine	GLY	1424.99
Threonine	TRE	357.27
Arginine	ARG	600.65
Alanine	ALA	11.15
Tyrosine	TYR	358.26
Cysteine	CYS	135.57
Valine	VAL	160.65
Methionine	MET	430.25
Phenylalanine	PHE	9.52
Isoleucine	ISO	251.19
Leucine	LEU	762.71
Lysine	LIS	714.83
AAA		367.78
NCAA		1017.84
PCAA		2109.63
HAA		2119.31
SCAA		565.82
EAA		4081.23
BCAA		1174.55

AAA: Aromatic amino-acid; NCAA: Negatively charged acids; PCAA: Positively charged acids; HAA: Total hydrophobic amino-acids; SCAA: Sulfuric containing amino-acid; EAA: Essential amino-acid; BCAA: Branch chain.

**Fig. 1:**
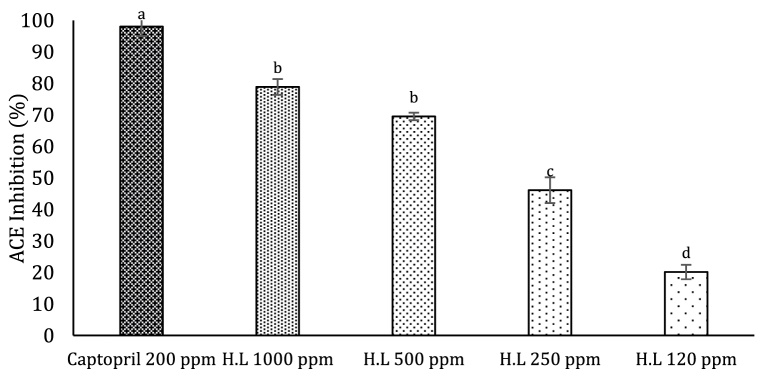
Percent angiotensin II-converting enzyme inhibition of Californian redworm hydrolysates as a function of concentration (100–120 ppm). Captopril was used as a positive control for ACE-II. Enzyme inhibition is expressed as per cent inhibition and the values represent the means of three independent experiments ± S.D. Different letters within the same parameter indicate significant differences (*p* < 0.05).

Fig. 1 presents the angiotensin-converting enzyme inhibitory activity (ACEI) results for the *E. fetida* hydrolysate (120–1000 ppm) and the captopril positive control (200 ppm). The ACEI percentage increases as the hydrolysate concentration increases, yielding a half-maximal inhibitory concentration (EC_50_) of 358 ppm (0.358 mg/mL). Research also shows that positively charged amino acids and aromatic amino acids contribute to peptide ACE inhibitory activity in different animal protein hydrolysates [18,38]. Toopcham et al. (2017) suggested that Leu and other branched chain (Ile and Val), hydrophobic, positively charged aliphatic amino acids (Lys and Arg) contribute to a higher ACE inhibitory potential. This value is below what is reported for rainbow trout enzymatic hydrolysates, which present an EC_50_ of 1.5 mg/mL [18]. Additionally, protein hydrolysates derived from round sardinella (*Sardinella aurita*), and Atlantic salmon (*Salmo salar*) by-products have reported ACEI EC_50_ values of 0.24 mg/mL and 0.74 mg/mL, respectively, with the *E. fetida* hydrolysate also falling within that range [39,40].

### 3.2 Organ bath vasodilation on isolated aortic rings

**Fig. 2:**
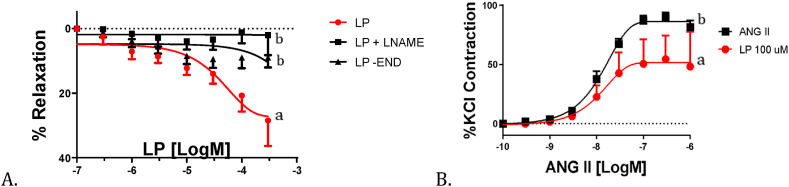
Vasodilator analysis in isolated organ (aortic rings) of rabbit A. Percentage of ring relaxation in the presence (LP) and absence of endothelium (LP-END) using L-NAME. B. Percentage of ring contraction with angiotensin II in the presence of worm hydrolysate (LP). Two-way analysis of variance followed by Student's t-test, significance level of *p-*value <0.05. Different letters within the same parameter indicate significant differences. KCl: potassium chloride; AngII: angiotensin-II.

Fig. 2 shows the vasodilator analysis on isolated rabbit organs (aortic rings). The relaxation percentage both in the presence of endothelium (LP) and absence of endothelium (LP-END) can be observed, and L-NAME was used as a control (Fig. 2A). The endothelium is a highly selective barrier and a metabolically highly active organ with a crucial role in vascular homeostasis [41], defined as a balance between a vasodilator and a vasoconstrictor state. The former is often associated with antioxidant, anti-inflammatory, and antithrombotic properties, and the latter is related to a prooxidant, proinflammatory, and prothrombotic state [42]. A maximum relaxation percentage of 38 % was achieved in the hydrolysate sample with endothelium (LP). Conversely, when the endothelium was removed, the relaxation percentage was much lower (approximately 5 %), with no statistically significant differences with L-NAME at specific points. This indicates that the vasorelaxation of *E. fetida* hydrolysate peptides is endothelium-dependent, which may be because the bioactive peptides regulate the nitric oxide synthase enzyme (NOSe), thus increasing the bioavailability of nitric oxide (NO) and giving it its hypotensive effect [43]. Likewise, this can be evidenced in Table 2, where the samples present statistically significant differences with p < 0.05.

**Table 2:** Sensitivity (pD2) and maximal responses (Emax) to L-Name with and without endothelium and to angiotensin-II in aortic rings.

Dilatation				
Agonist	pD_2_	p	%Emax	p
LP	4.48 ± 0.08	0.04	28.45 ± 8	0.018
LP + Sin endothelin	5.7 ± 0.1		9.408 ± 3.2	
LP + L-name	5.78 ± 0.18		3.924 ± 5.3	
**Contraction**				
**Agonist**	**pD**_**2**_	**p**	**%Emax**	**p**
Angiotensin 2	7.82 ± 0.03	0.05	89.86 ± 5.2	0.008
Angiotensin 2 + LP	7.84 ± 0.07		54.38 ± 21.8	

All the values are expressed as mean ± SEM (n = 5). Emax, maximal effect expressed as a percentage of the response induced; pD2 -log one-half Emax. *p* < 0.05, Student's t-test.

Oxidative stress is associated with hypertension, as reduced concentrations of nitric oxide cause vasoconstriction, generating endothelial dysfunction. Due to this, having access to a supply of dietary antioxidants is fundamental, and it shows the relationship between antioxidant agents and the inhibition of arterial hypertension [20,23]. However, this dysfunction may also be associated with elevated renin-angiotensin-aldosterone system activity, increasing free radical production. Thus, there is no single cause for high blood pressure [24]. Fig. 2B shows the ring contraction percentage caused by angiotensin II in the presence of earthworm hydrolysate (LP). Ang II can contract the aorta by up to 90 % due to its vasoconstrictor capabilities [42]. However, upon complete treatment with earthworm hydrolysate (LP), there was a decrease in the contraction caused by Ang II. This effect is explained and illustrated in Fig. 1 with the angiotensin-converting enzyme inhibition analysis. The contraction presented statistically significant differences with a value of p < 0.05 between the treatments with and without the *E. fetida* hydrolysate, which ratifies the effect of LP on reducing contraction when caused by angiotensin II. These results align with those obtained for *in vitro* ACE inhibition (Fig. 1).

### 3.3 Acute hypotensive activity in rats

**Fig. 3:**
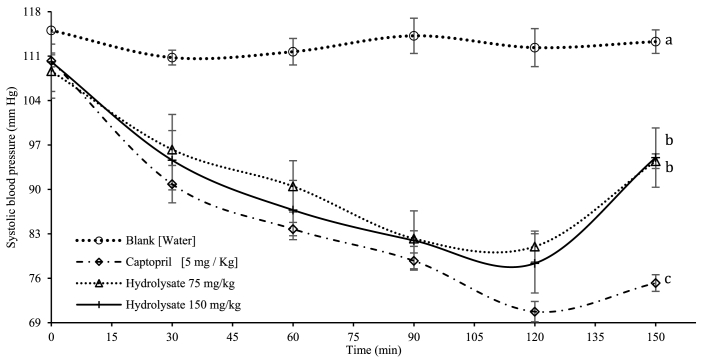
Hypotensive evaluation of enzyme hydrolysate on normotensive rats. Captopril and water were used as a positive and negative (Blank) control respectively. Values are expressed as means ± standard deviation (SD; n = 5). Different letters within the same parameter indicate significant differences (*p* < 0.05).

Fig. 3 shows the hypotensive effect of the *E. fetida* hydrolysate applied in rats at two concentrations (75 and 150 mg/kg) for 150 min. The same hypotensive effect was present at both hydrolysate concentrations when applied to the animals. Likewise, up to the 90 min timepoint, there were no significant differences between the hydrolysates and the control (captopril 5 mg/kg). The maximum peak of hypotension was achieved at 120 min, with a percentage reduction of 32 %. From this minute on, blood pressure began increasing to return to a normotensive state. When comparing the results with those of the control (captopril), the latter delivers a higher maximum reduction percentage (37 %) in the same amount of time the hydrolysate does, which is evidence that the hydrolysate is effectively absorbed in the study animals, reducing blood pressure significantly [44]. These results are relevant when compared to those reported by Ref. [45], who administered a dose of 200 mg/kg of smooth-hound viscera hydrolysate and achieved a reduction in systolic pressure by up to 10 %. Separately, Hayes et al. (2016) reduced systolic pressure by 15 mmHg after 2 h using boarfish hydrolysates. However, the maximum reduction peaks were reached at 8 h with 36.91 %, which is similar to what is reached with the *E. fetida* hydrolysate in 2 h [46].

### 3.4 Chronic antihypertensive activity in rats

**Fig. 4:**
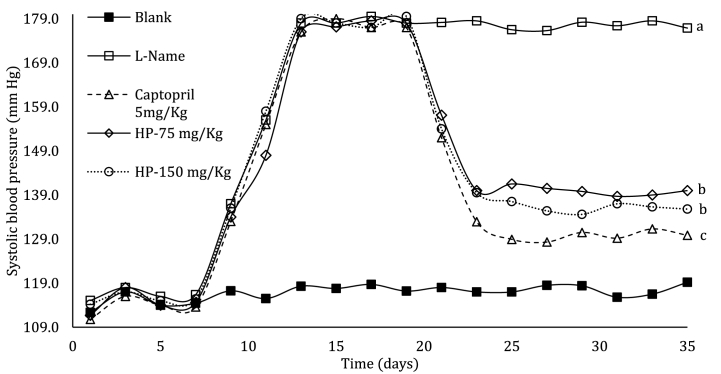
Evaluation of antihypertensive activity on rats with induced hypertension. Captopril and water were used as a positive and negative (Blank) control respectively. Values are expressed as means ± standard deviation (SD; n = 5). Statistical evaluation was carried out and compared with the negative control group (water). Different letters within the same parameter indicate significant differences (*p* < 0.05).

For the chronic antihypertensive assay in hypertensive rats, two doses of the *E. fetida* hydrolysate (75 and 150 mg/kg) were evaluated using captopril 5 mg/kg as a positive control and saline solution as a blank. Fig. 4 shows how systolic blood pressure reduced significantly starting on day 20, which is when we initiated treatment with the hydrolysate and controls. From day 23 onwards, the pressure stabilized at 138, 134, and 130 mmHg for hydrolysate 75 mmHg, hydrolyzate 150 mmHg, and captopril, respectively. The antihypertensive effect of the hydrolysate was similar to that of the positive control, thus showing the potential that these hydrolysates have for possible hypertension treatment. Equivalent results have been reported with the administration of skate skin gelatin hydrolysates at 1000 mg/kg on spontaneously hypertensive rats, presenting maximum reduction at 130 mmHg after 20 days [47]. These results are akin to those found using the *E. fetida* hydrolysate at a dose approximately seven times lower (150 mg/kg). On the other hand [43], studied the effect of myosin hydrolysates (*Trichiurus lepturus*) for one month, implementing two doses (400 and 100 mg/kg) and reaching values of 140 mmHg with the higher doses, without presenting statistically significant differences with the control (captopril 5 mg/kg). On comparing these results with those obtained in the present study, the decrease in blood pressure was greater at a lower dose of *E*. *fetida hydrolysate*, highlighting its antihypertensive potential.

## 4 Conclusion

The results of this study showed that red Californian earthworm (*Eisenia fetida*) proteins can be used to produce hydrolysates with potent ACE-II inhibitory peptides. Likewise, at the *ex vivo* level, it reduces contraction to 50 % with 100 μM of the hydrolysate and *in vivo*, the hydrolysate administered at 150 mg/kg significantly reduced systolic pressure, and the effect was sustained over time. This last statement explains why it can control and regulate arterial hypertension through different synergetic routes of action (inhibition of the angiotensin-converting enzyme and regulation of nitric oxide production). These results advocated the use of this multiple assessment approach to evaluate the antihypertensive potential of a hydrolysate or peptide.

These results suggest that ACE inhibitory hydrolysate from red Californian earthworm (*Eisenia fetida*) protein could be potential candidates to develop functional foods, nutraceutical and pharmaceutical products against hypertension.

## 5 Limitations

Identifying the peptide composition of protein hydrolysates remains a formidable challenge, necessitating the development of improved methods for accurate identification. Furthermore, there exists a notable deficiency in our understanding of the structure-function relationships governing peptide activities. Thus, additional comprehensive studies are imperative to enhance our comprehension in this domain. The incorporation of bioactive peptides into various applications is hindered by undesirable off-flavors, particularly in the context of oral ingestion. As a result, it is imperative to devise suitable strategies for either eliminating or masking these off-flavors. Lastly, the exploration of the potential biological activities and health benefits of earthworm peptides is currently limited by the scarcity of clinical human studies. To elucidate the physiological significance of earthworm peptides, a more extensive body of human studies is warranted.

### Ethics statement

The Ethics Committee for Animal Experimentation of the University of Antioquia in the minutes of session number 136 (extraordinary) of November 17, 2020, and the Bioethics Committee of the 10.13039/100019348University of Tolima in session number 06 of 2019 granted approval for the experiments performed in this manuscript.

## Additional information

No additional information is available for this paper.

## Data availability statement

Publicly available datasets were analyzed in this study.

## CRediT authorship contribution statement

**Yhoan S. Gaviria G:** Writing – review & editing, Writing – original draft, Methodology, Investigation, Formal analysis, Data curation, Conceptualization. **Carlos M. Guerra:** Methodology, Conceptualization. **José E. Zapata M:** Writing – review & editing, Writing – original draft, Project administration, Funding acquisition, Conceptualization.

## Declaration of competing interest

The authors declare that they have no known competing financial interests or personal relationships that could have appeared to influence the work reported in this paper.
